# Immunogold electron microscopic evidence of *in situ *formation of homo- and heteromeric purinergic adenosine A_1 _and P2Y_2 _receptors in rat brain

**DOI:** 10.1186/1756-0500-3-323

**Published:** 2010-11-29

**Authors:** Kazunori Namba, Tokiko Suzuki, Hiroyasu Nakata

**Affiliations:** 1 Department of Molecular Cell Signaling, Tokyo Metropolitan Institute for Neuroscience, 2-6 Musashidai, Fuchu, Tokyo 183-8526, Japan; 2 Otolaryngology/Lab. of Auditory Disorders, National Institute of Sensory Organs National Tokyo Medical Center, 2-5-1 Higashigaoka, Meguro, Tokyo 152-8902, Japan; 3 Department of Cellular Signaling, Graduate School of Pharmaceutical Sciences, Tohoku University, Aoba 6-3, Aramaki, Aoba-ku, Sendai 980-8578, Japan

## Abstract

**Background:**

Purines such as adenosine and ATP are now generally recognized as the regulators of many physiological functions, such as neurotransmission, pain, cardiac function, and immune responses. Purines exert their functions via purinergic receptors, which are divided into adenosine and P2 receptors. Recently, we demonstrated that the G_i/o_-coupled adenosine A_1 _receptor (A_1_R) and G_q/11_-coupled P2Y_2 _receptor (P2Y_2_R) form a heteromeric complex with unique pharmacology in co-transfected human embryonic kidney cells (HEK293T). However, the heteromeric interaction of A_1_R and P2Y_2_R *in situ *in brain is still largely unknown.

**Findings:**

In the present study, we visualized the surface expression and co-localization of A_1_R and P2Y_2_R in both transfected HEK293T cells and in rat brain by confocal microscopy and more precisely by immunogold electron microscopy. Immunogold electron microscopy showed the evidence for the existence of homo- and hetero-dimers among A_1_R and P2Y_2_R at the neurons in cortex, cerebellum, and particularly cerebellar Purkinje cells, also supported by co-immunoprecipitation study.

**Conclusion:**

The results suggest that evidence for the existence of homo- and hetero-dimers of A_1_R and P2Y_2_R, not only in co-transfected cultured cells, but also *in situ *on the surface of neurons in various brain regions. While the homo-dimerization ratios displayed similar patterns in all three regions, the rates of hetero-dimerization were prominent in hippocampal pyramidal cells among the three regions.

## Background

The adenosine A_1 _receptor (A_1_R) is known to regulate Ca^2+^/K^+ ^channels, adenylate cyclase, and phospholipase C by coupling to G_i/o _proteins [1]. In hippocampal astrocytes, P2Y_1_R- and P2Y_2_R-mediated Ca^2+ ^responses differentially show two forms of activity-dependent negative feedback of synaptic transmission via the phospholipase C beta-IP_3 _pathway [2]. Today, the homo- or hetero-dimers of many kinds of GPCRs have been reported [3]. We previously demonstrated that A_1_R associates with P2Y_1_R in co-transfected HEK293T cells and in rat brain homogenates, whereby a P2Y_1_R agonist stimulates A_1_R signaling via G_i/o _[4,5]. Furthermore, in HEK293T cells co-transfected with A_1_R and P2Y_2_R, the heterodimers display synergistic increases in Ca^2+ ^signaling, whereby simultaneous activation of the two receptors attenuates A_1_R signaling via G_i/o_, but synergistically enhances P2Y_2_R signaling via G_q/11 _[6]. Also, the simultaneous activation of endogenous A_1_R and P2Y_2_R in DDT1MF-2 cells synergistically increases translocation of protein kinase C [7]. Because A_1_R are widely expressed in brain [5], it is likely that these receptors also associate directly *in situ*; however, direct evidence of their dimerization or precise co-localization in brain has yet to be demonstrated. The aim of the present study is to determine whether A_1_R and P2Y_2_R associate with each other in rat brain by co-immunoprecipitation and looking for receptor complexes via immunogold electron microscopy (IEM).

## Methods

### Double immunostaining of A_1_R/P2Y_2_R in HEK293T cells and rat brain sections

Double immunostaining using anti-HA 3F10 mAb rat antibody (anti-HA) and anti-Myc 9E10 mAb mouse antibody (anti-Myc) in HA-A_1_R and Myc-P2Y_2_R-co-transfected HEK293T cells were performed as previously described [6]. Cells were washed and then stained with Alexa 568-conjugated goat anti-rat IgG antibody (1:200, Invitrogen, Carlsbad, CA) for A_1_R or Alexa 488-conjugated goat anti-mouse IgG antibody (1:200, Invitrogen) for P2Y_2_R. The characterization of antibodies for rat brain sections was previously reported, although the rabbit polyclonal anti-P2Y_2_R antibody (anti-P2Y_2_R; 1 μg/ml, Alomone Labs, Jerusalem, Israel) was used instead of the rabbit polyclonal anti-P2Y_1_R antibody [5,8].

### Immunoprecipitation and western blotting of rat brain homogenates

Eight-week-old male Wistar rats were decapitated under anesthesia (Nembutal; 30 mg/kg i.v.), and cortical, hippocampal, and cerebellar tissues were dissected out. The tissues were homogenized with a Polytron homogenizer in 50 mM Tris-acetate, pH 7.4, containing a protease inhibitor cocktail (Roche Applied Science, Manheim, Germany), and the resulting cell suspensions were centrifuged at 30,000 × g for 30 min at 4°C. The pellets were solubilized in ice-cold lysis buffer (50 mM Tris-HCl, pH 7.4, 1% Triton X-100, 300 mM NaCl and a protease inhibitor cocktail) for 60 min at 4°C. The mixture was centrifuged at 18,500 × g for 20 min at 4°C, and the supernatant pre-cleared with Protein G-Sepharose™4 Fast Flow (Amersham Bioscience, Piscataway, NJ). The lysate was incubated with rabbit polyclonal anti-A_1_R antibody (anti-A_1_R; 1 μg/ml, Sigma-Aldrich, St. Louis, MO) for 60 min at 4°C. Protein G-Sepharose was added to the mixture, and the incubation continued for an additional 120 min. Protein G-Sepharose was recovered by centrifugation and washed three times with lysis buffer. Immunoprecipitates were eluted with SDS-PAGE sample buffer, resolved by 12% SDS-PAGE, and electrotransferred to nitrocellulose membranes. Receptors on the blot were detected using anti-A_1_R or anti-P2Y_2_R, followed by horseradish peroxidase-conjugated goat anti-rabbit IgG secondary antibody (Sigma-Aldrich). The reactive bands were visualized with enhanced chemiluminescent substrates (SuperSignal West Pico, Pierce, Rockford, IL).

### Pre-embedding immunogold electron microscopy (IEM) of transfected HEK293T cells

HEK293T cells expressing HA-A_1_R and Myc-P2Y_2_R were fixed with 4% PFA, and permeabilized with 0.25% Triton X-100. Cells were incubated with anti-HA and anti-Myc for 3 h at 4°C. After washing with PBS, cells were incubated with 10-nm gold particle-conjugated goat anti-rat IgG antibody (rat IgG-10, 1:1000, BBI International, Lakewood, CO) and 5-nm gold particle-conjugated goat anti-mouse IgG antibody (mouse IgG-5, 1:1000, BBI International) for 4 h at 4°C. After washing, the cells were fixed with 2.5% glutaraldehyde in 0.15 M sodium cacodylate, pH 7.4 for 2 h, washed, and postfixed with 1% osmium tetroxide for 4 h at room temperature. The cells were then dehydrated and embedding resin (Epon 812; NISSIN EM, Tokyo, Japan). Specimens were observed with an H7500 electron microscope (Hitachi, Japan). We quantified the gold staining as follows: The gene-transfected HEK293T cells with the highest numbers of total immuno-reacted gold particles were defined as 100% labeling. Because the co-transfected HEK293T cells that displayed unique pharmacology in our previous study [6] exhibited more than 20% hetero-dimeric gold particles, we used this number as a threshold in the current study. Thus, cells with more than 20% hetero-dimeric particles were defined as being "significantly stained", and those with 20% or less were defined as "not significantly stained".

### Post-embedding immunogold electron microscopy of brain tissues

Dissected brain tissues were cut into 1.0 mm^3 ^blocks that were then incubated with lead (II) acetate (Sigma-Aldrich) buffer for 1 h at room temperature, dehydrated through a series of graded ethanol, and embedded in LR-white (NISSIN EM). Ultra thin sections (40 nm) were mounted on 200-mesh nickel grids (NISSIN EM) and incubated in PBS containing 1% BSA for 10 min. After immunostaining with primary antibodies, each specimen was incubated with mouse IgG-5- and IgG-10-nm gold particle-conjugated goat anti-rabbit IgG antibody (rabbit IgG-10) for 6 h at 4°C. For controls, transfected HEK293T cells were embedded with LR-white under the same conditions as described above. After incubation at 4°C for 12 h with anti-HA (10 μg/ml) and anti-Myc (10 μg/ml), samples were washed with 1% BSA/PBS. After incubation with gold particle-conjugated secondary antibodies for 6 h at 4°C, sections were stained with uranyl acetate for 10 min. "Significant heteromeric staining" was defined as more than 20% of the total number of immuno-reacted gold particles at the cell surface occurring in heteromeric clusters.

### Comparison of the numbers of monomers, homo-dimers, and hetero-dimers

**Figure 1:**
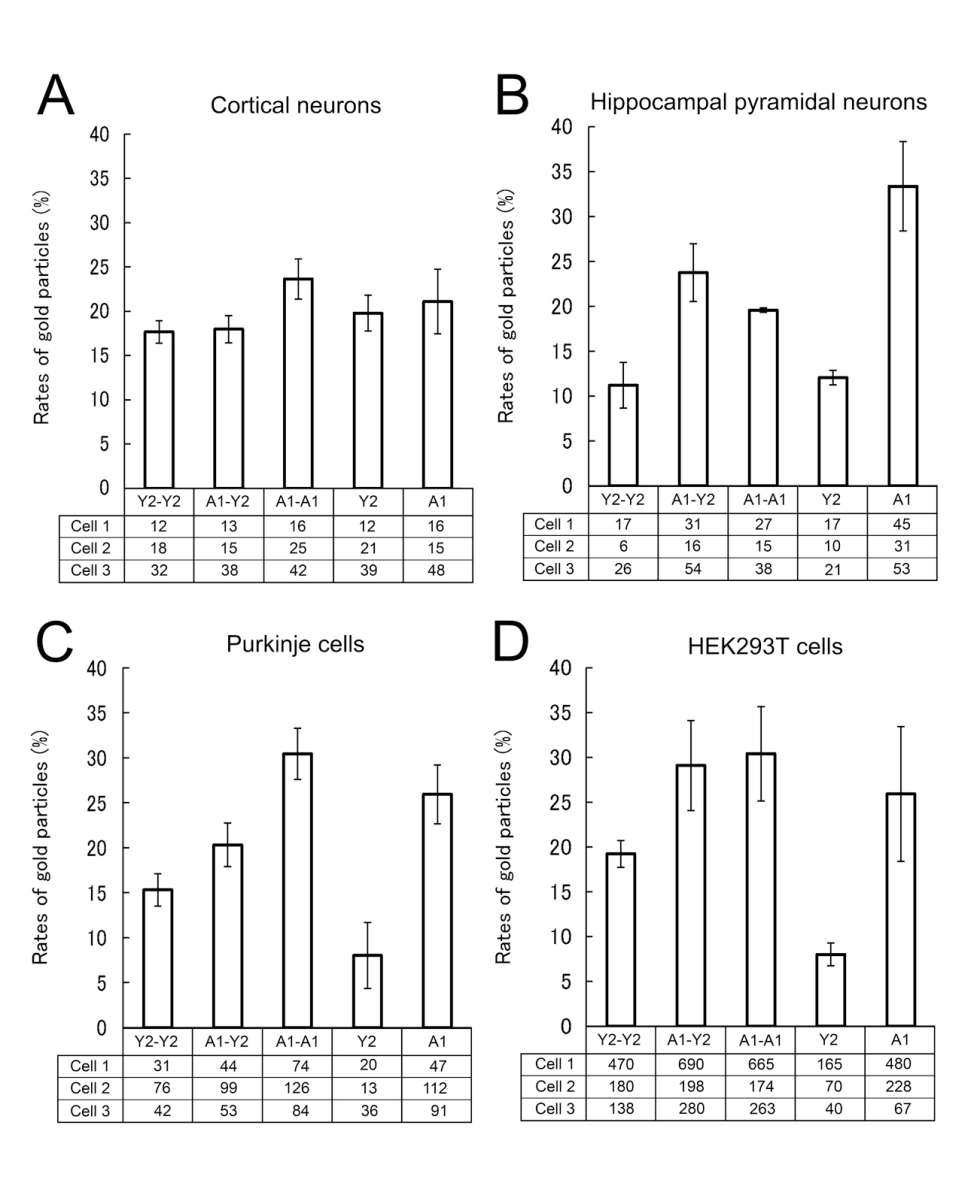
**Bar graphs comparing the relative distributions of A_1_R(A1)- and P2Y_2_R(Y2)-immunoreactive elements in each brain region (A-C) and in transfected HEK293T cells (D)**. The P2Y_2_R-P2Y_2_R, A_1_R-A_1_R and A_1_R-P2Y_2_R dimers are indicated by Y2-Y2, A1-A1 and A1-Y2, respectively. Total number of immunoreactive gold particles on the cell surface was defined as 100%. Each column represents the average frequency (± SD) from three cells. Raw data are shown in the tables under the graphs. Data are means of three independent experiments.

The numbers of immunogold particles at the cell surface of each cell type were determined. We defined single particles located independently as monomers (A_1_R and P2Y_2_R in Figure 1), complexes composed of clusters of the same-sized gold particles as "homo-dimers" (A_1_R-A_1_R or P2Y_2_R-P2Y_2_R in Figure 1), and those of different sized gold particles as "hetero-dimers" (A_1_R-P2Y_2_R in Figure 1). Separate calculations were made of particles in cortical neurons (Figure 1A), hippocampal pyramidal neurons (Figure 1B), and Purkinje cells (Figure 1C); gold particles were counted in three cells in each region. We also counted immunogold particles in co-transfected HEK293T cells (please see above, and Figure 1D). The total number of immunoreactive gold particles on each cell surface was defined as 100%. From a total of 12 photos from each brain area (i.e., 36 photos) and from transfected cells that were reacted under the same conditions as the brain sections for each immunostaining, the three photos of each specimen containing whole cells were selected randomly for comparison.

## Results

### Co-localization of A_1_R and P2Y_2_R in transfected HEK293T cells

**Figure 2:**
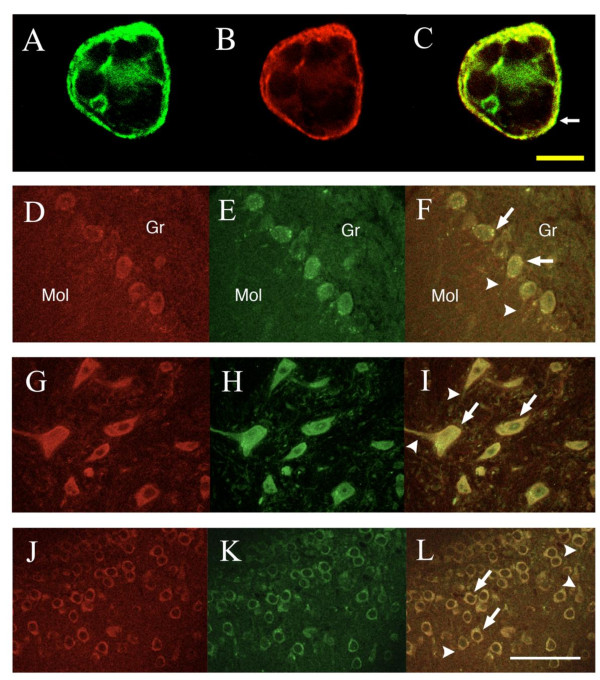
**Co-localization of A_1_R and P2Y_2_R**. *A-C*. Confocal images of double immunostained Myc-P2Y_2_R (A; green), HA-A_1_R (B; red), and their merge (C; yellow) in co-transfected HEK293T cells. The co-localization of HA-A_1_R and Myc-P2Y_2_R is evident at the cell surface membrane (small arrow). *D-L*. Confocal images of double immunofluorescence staining in several rat brain regions. P2Y_2_R (D, G, J; red) and A_1_R (E, H, K; green) immunoreactivities were detected in Purkinje cells (D-F), cerebellar nuclei (G-I), and hippocampal CA3 pyramidal cells (J-L). Co-localizations of A_1_R and P2Y_2_R (F, I, L; yellow) were detected in the soma (large arrows) of all tissues, in dendrites of the Purkinje cells, and in neurons of the cerebellar nuclei (arrowheads). Yellow bar indicates 500 μm (A-C) and white bar indicates 100 μm (D-L). Mol: cerebellar molecular layer, Gr: cerebellar granule cell layer. Fluorescent images were collected via confocal laser scanning microscopy (Zeiss LSM410, Carl Zeiss, Oberkochen, Germany) each 10-μm optical slice consisted of a stack of 20 0.5-μm thick sections. Serial optical sections were recorded using an air objective lens of (40×, numerical aperture; 0.6).

The co-localization of A_1_R and P2Y_2_R in co-transfected HEK293T cells was examined by double immunostaining of HA-A_1_R and Myc-P2Y_2_R as a comparison experiment for the localization of these receptors in brain tissues (Figure 2). Both receptors were localized mainly on cell surface and cytosolic membranes, but not in the nucleus (Figure  2A, B). Merged images showed their co-localization mainly in cell membranes (Figure 2C). No signals were observed in non-transfected HEK293T cells, indicating that the immunoreactivity observed in Figure 2 was specific to the expressed receptors (data not shown). These results suggest that both receptors were expressed on cell membranes.

### Immunohistochemical studies in rat brain

We examined the expression of A_1_R and P2Y_2_R in brain using immunohistochemical analyses (Figure 2). The specificity of the antibodies against A_1_R and P2Y_2_R was confirmed by the immunocytochemistry of recombinant receptor-expressing cell lines, i.e. antibodies used in this study showed no cross-labeling in A_1_R- and P2Y_2_R-transfected HEK293T cells (data not shown). Prominent staining of A_1_R and P2Y_2_R were observed especially in Purkinje cells (Figure 2D-F), interposed cerebellar nuclei (Figure 2G-H), and hippocampal pyramidal cells (Figure 2J-L). Comparatively high immunoreactivities were also detected in the piriform cortex, amygdala, hypothalamus, and brainstem (data not shown). Their expressions were mainly restricted to cell bodies and neuronal dendrites. Importantly, co-localization of A_1_R and P2Y_2_R in the cerebellum was observed in cell bodies, except in the nuclear region, in the Purkinje cells and those of the interposed cerebellar lobule nucleus (Figure 2D-I). In the hippocampal region, pyramidal cell bodies, especially the cell surface membranes, in CA1, CA2, CA3, and the dentate gyrus (CA3; Figure 2J-L, others; data not shown) were intensely stained for both A_1_R and P2Y_2_R. Similar staining patterns were seen in cell bodies of neurons in the cerebral cortex (data not shown).

### Co-immunoprecipitation of A_1_R and P2Y_2_R from rat brain

**Figure 3:**
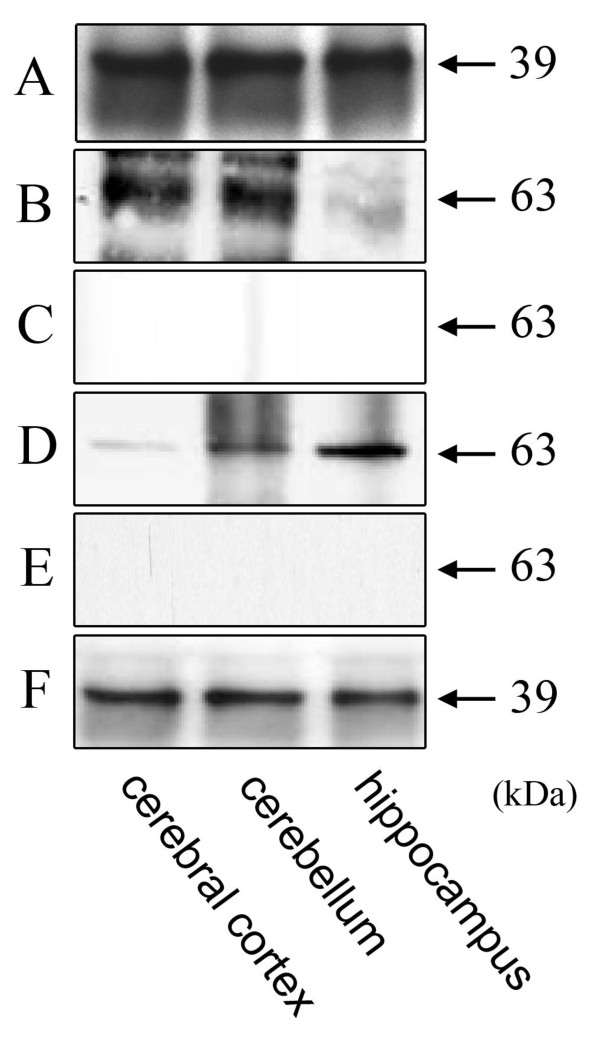
**Co-immunoprecipitation of A_1_R from cerebral cortex (lane 1), cerebellum (lane 2), and hippocampus (lane 3)**. Immunoblotting analyses of extracts of rat brain with anti-A_1_R (A), anti-P2Y_2_R (B), and anti-P2Y_2_R with the control peptide of P2Y_2_R (C). Membrane extracts from each region were immunoprecipitated with anti-A_1_R, and analyzed by immunoblotting with anti-P2Y_2_R (D), anti-P2Y_2_R with the control peptide of P2Y_2_R (E), anti-A_1_R (F). No bands were seen in C, E demonstrating the specificity of the antibodies. It was confirmed that immunoprecpitation without primary antibody resulted in no detectable receptor bands in the immunoblotting (data not shown). Approximate molecular masses are shown in kDa.

Next, we examined whether A_1_R and P2Y_2_R are associated with one another in several brain regions using immunoprecipitation with anti-A_1_R followed by immunoblotting with both A_1_R and P2Y_2_R antibodies (Figure 3). A_1_R and P2Y_2_R immunoreactivities were present in all three rat brain regions examined (Figure  3A, B, F). Moreover, in these same regions, anti-A_1_Rs were capable of co-precipitating P2Y_2_R (Figure 3D), indicating that A_1_R and P2Y_2_R are associated with one another in rat cortex, cerebellum, and hippocampus. The absence of these immunoreactive bands in the presence of anti-P2Y_2_R antigen peptides (Figure  3C, E) is evidence of their specificity of the antibodies. The specificity of the anti-A_1_R was confirmed by immunocytochemistry of mock-transfected HEK293T cells, and no specific band was detected (data not shown).

### Immunogold electron microscopic observations of HA-A_1_R and Myc-P2Y_2_R expressed in HEK293T cells

**Figure 4:**
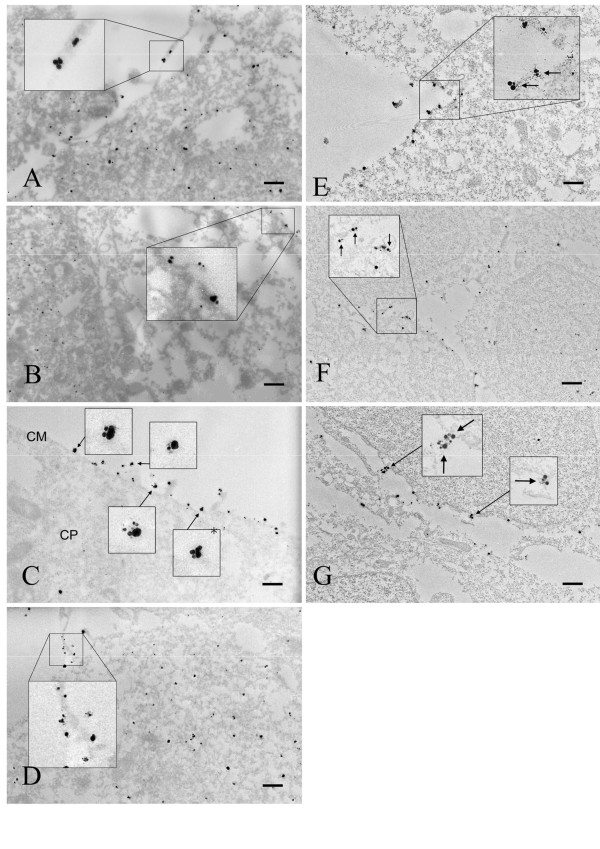
**Immunogold electron microscopy of A_1_R and P2Y_2_R visualized using nanogold particles in transfected HEK293T cells (A-D) and rat brain (E-G)**. A: Localization of HA-A_1_R (large particles) detected with anti-HA in HA-A_1_R-transfected HEK293T cells. B: Localization of Myc-P2Y_2_R (small particles) detected with anti-Myc in Myc-P2Y_2_R-transfected HEK293T cells. C: Anti-HA and anti-Myc immuno-localization of HA-A_1_R and Myc-P2Y_2_R in co-transfected HEK293T cells. D: HA-A_1_R-transfected HEK293T cells incubated with both anti-HA and anti-Myc. E-G: Localization of A_1_R and P2Y_2_R in cortical pyramidal cells (E), Purkinje cells (F), and hippocampal pyramidal cells (G) detected with both anti-A_1_R and anti-P2Y_2_R. Arrows indicate two adjacent receptors on the cell membrane. Bars represent 100 nm. CM, cell membrane; CP, cytoplasm.

The immunogold particles were localized singly or in clusters, indicating that both HA-A_1_R and Myc-P2Y_2_R form monomers and homo-dimers. Specificities of the gold-labeled anti-HA and anti-Myc were demonstrated by incubating A_1_R-transfected HEK293T cells with a mixture of both antibodies, and showed that only A_1_R-labeled particles were present (Figure 4D). No significant immunoreactivity was detected with both anti-HA and anti-Myc in mock-transfected HEK293T cells or with only secondary antibodies (no primary antibodies) in HA-A_1_R-transfected HEK293T cells (data not shown). Also, when Myc-P2Y_2_R-transfected HEK293T cells were incubated with both anti-HA and anti-Myc, single particles (monomers) were scattered all over the cells, whereas co-localized, equal-sized particles of Myc-P2Y_2_R (homo-dimers) were only occasionally seen (data not shown). In HEK293T cells co-transfected with both HA-A_1_R and Myc-P2Y_2_R, clusters of different-sized particles were observed mainly at the cell surface (Figure 4C) might be suggestive that they form heteromeric complexes.

### Immunogold electron microscopic observations of A_1_R and P2Y_2_R expressed in rat brain

We incubated post-embedded, primary antibody-stained rat brain tissues with secondary antibodies labeled with mouse IgG-5 for A_1_R and rabbit IgG-10 for P2Y_2_R. As negative controls, tissues were stained with only secondary antibodies conjugated with different sized gold particles; no significant immunoreactivities were observed under the experimental conditions used in this study (data not shown). As in the transfected HEK293T cells, we observed clusters of different-sized gold particles at cytoplasmic membranes in cell bodies, indicating the presence of heteromeric complexes of endogenous A_1_R and P2Y_2_R in rat brain (Figure 4E-G). Significant immunoreactivity was detected in Purkinje cells (Figure 4F) and hippocampal pyramidal cells (Figure 4G). Hetero- and homo-dimers were detected in significant numbers at the cell surface in both transfected HEK293T cells and native brains.

### Comparison of the frequencies of monomers, homo-dimers, and hetero-dimers

We counted gold particles on the surfaces of cells in the cortex, cerebellum, and hippocampus and classified them as monomers (A_1_R or P2Y_2_R), homo-dimers (A_1_R-A_1_R or P2Y_2_R-P2Y_2_R), or hetero-dimers (A_1_R-P2Y_2_R). While the homo-dimerization ratios (A_1_R-A_1_R/P2Y_2_R-P2Y_2_R) displayed similar patterns in all three regions (Figure 1A-C), the rates of hetero-dimerization were prominent in hippocampal pyramidal cells among the three regions.

## Discussion

The present study provides the first detailed evidence of an interaction between endogenous A_1_R and P2Y_2_R in brains using co-immunoprecipitation and IEM. The homo-dimerization of A_1_R was previously analyzed in our laboratory by computational prediction, co-immunoprecipitation, and BRET analysis [9]. In the present study, we might suggest the existence of homo-dimers (A_1_R-A_1_R and P2Y_2_R-P2Y_2_R) using IEM. Very interestingly, the percentage of A_1_R homo-dimers was higher than that of P2Y_2_R in both rat brain and transfected HEK293T cells (Figure 1). By contrast, the ratios of heteromeric gold-particle clusters were different in the cortex, hippocampus, and cerebellum. Importantly, both homo-dimeric and hetero-dimeric gold-particles were much fewer at inner cytoplasmic membranes than at the cell surface (data not shown). In general, most GPCRs dimers have been observed on the cell surface [10,11]. Total numbers of hetero-dimers observed on the cell surface and in the cytoplasm were obviously different (data not shown) and may reflect the process of receptor maturation and association of the A_1_R-P2Y_2_R complex.

In the hippocampal region, the strong presence of hetero-dimers coincided with the relative signal intensity of the co-immunoprecipitation band (Figure 3D lane 3). In the previously reported electron microscopic analysis of A_1_R and P2Y_1_R co-localization in hippocampus, the A_1_R density was relatively higher than that of P2Y_1_R at the presynaptic membrane [12]. They suggested that the hetero-dimerization or cross-talk of A_1_R and P2Y_1_R is involved in regulation of glutamate release. The relative distributions of immunoreactivities for GABA_B _R2 and GABA_B _R1 were also different in the basal ganglia and globus pallidus/substantia nigra, which suggests the possible co-existence and hetero-dimerization of two types of receptors at various pre-/postsynaptic sites [13]. From the present study, it can be speculated that the A_1_R/P2Y_2_R hetero-oligomer might be responsible for down regulation, via hippocampal Ca^2+ ^secretion, of synaptic functions [14]. The abundant formation of A_1_R/P2Y_2_R hetero-oligomers in hippocampus revealed in this study supports the idea that the unique signal transduction generated by hetero-dimerization, including the enhancement of Ca^2+ ^signaling via G_q/11_, observed in transfected cells also occurs in hippocampus.

## List of abbreviations

GPCR: G protein-coupled Receptor; A_1_R: A_1 _adenosine receptor; P2Y_1_R: P2Y_1 _purinergic receptor; P2Y_2_R: P2Y_2 _purinergic receptor; IEM: immunogold electron microscopy

## Competing interests

The authors declare that they have no competing interests.

## Authors' contributions

KN carried out all experiments, prepared the figures and drafted the manuscript. TS assisted immunostaining experiment and in manuscript revising. NH was responsible for experimental design and revised and polished the manuscript. All authors have read and approved final manuscript.
